# A dataset of fluvial fish richness metrics for stream reaches across the conterminous U.S.

**DOI:** 10.1038/s41597-026-07177-4

**Published:** 2026-08-10

**Authors:** Daniel J. Wieferich, Hao Yu, Mark Wiltermuth, Wesley M. Daniel, Jared A. Ross, Arthur R. Cooper, Dana M. Infante

**Affiliations:** 1 U.S. Geological Survey, Science Analytics and Synthesis, Ann Arbor, MI USA; 2 Michigan State University, East Lansing, MI USA; 3 U.S. Geological Survey, Science Analytics and Synthesis, Lakewood, CO USA; 4 U.S. Geological Survey, Wetland and Aquatic Research Center, Gainesville, FL USA

**Keywords:** Biogeography, Biodiversity

## Abstract

Fluvial fishes, those primarily inhabiting rivers and streams, are facing substantial threats in their native ranges from habitat degradation which contributes to decreases in richness and abundance of many species. Because distributions of fluvial species are often not well developed across large spatial scales, the ability to understand the consequences of threats is limited. Species distribution models were recently developed for roughly one third of freshwater fish species in the United States typically found in fluvial habitats. To highlight locations of potential value for species management and conservation, we developed 18 fluvial fish species richness metrics. Metrics were developed using results of 415 readily available species distribution models that indicate likelihood of species presence on approximately 2.3 million stream reaches of the conterminous United States as characterized by the National Hydrography Dataset Plus Version 2.1. Specific metrics fall into groups including the full set of 415 modeled species, migratory species, species of greatest conservation need, and game fish species, providing utility for varied conservation and management priorities of fluvial fishes.

## Background & Summary

The United States (U.S.) is home to one of the most diverse assemblages of freshwater fishes in the world, with roughly 1,200 native species inhabiting rivers, streams, lakes, and wetlands^1,2^. Consequently, fluvial fishes, those primarily inhabiting rivers and streams, are facing substantial threats in their native ranges from invasive species introduction and habitat degradation such as altered hydrology, fragmentation and water pollution^3–5^. Decreasing species richness and abundance of fluvial fishes have been observed in many regions^6,7^, which can directly influence the economy through reductions in commercial and recreational fishing opportunities, and can further impact the environment in several ways such as shifting food webs, ecosystem resilience, and water quality.

Accurately describing fluvial fish distributions can enhance fishery resources management and conservation, as well as fish habitat restoration. Additionally, an ensemble of multiple species distributions can provide information about potential diversity and community make-up which are often emphasized throughout conservation and restoration efforts. Fisheries studies providing fish occurrence and distribution information, however, often focus on a single species, sample a small fraction of existing fluvial habitats, and are conducted at local to regional spatial scales^8^. These characteristics of studies lead to a lack of sufficient fish occurrence data for many species to represent the extensive number of fluvial habitats they inhabit within their native ranges. In addition, fluvial fish sampling efforts in different regions often differ with respect to methods and designs, further complicating the interpretation of available distribution data when making decisions about conservation and habitat management.

Species distribution models (SDMs) can be used for predicting where species are likely to occur, helping to bridge the gap between available records of occurrences using broader range-wide environmental information^9^. By using environmental data and known occurrences to estimate species occurrence probabilities in unsampled areas, SDMs can provide a more complete understanding of a species’ potential geographic range. The distributional predictions derived from SDMs can help focus monitoring, management, and conservation efforts by providing more relevant information than field sampling data alone. A summary of more detailed conservation and restoration applications using SDMs were highlighted in a recent review paper by Rathore and Sharma^10^. A few examples include evaluation of current and proposed protected areas, priority area identification, and species richness and abundance assessments. With similar purpose, we previously developed SDMs for 415 fluvial fish species by combining known fish occurrence records at 35,918 locations with 22 environmental variables known to influence fish habitat^11,12^. The SDMs we developed targeted fluvial fishes within their native ranges across the conterminous U.S. and were limited to species having sufficient occurrence data to pass model evaluations using a tenfold cross-validation procedure. For every stream reach within the range of a species, the corresponding SDM provided a likelihood of the species being present. Within this dataset, stream reaches are considered the smallest segments of streams represented in the 1:100,000 scale National Hydrography Dataset Plus Version 2.1^13^. These stream reaches represent a section of a stream or river along which similar hydrologic conditions exist. This effort covered approximately one third of all native freshwater fish species in the conterminous U.S., which to our knowledge represents the largest effort of its type for freshwater fishes based on its geographic and taxonomic scope.

Our inventory of SDMs that cover a large portion of all native freshwater species in the Conterminous U.S. and have information at the reach level, presents a unique opportunity to characterize fish richness patterns to support scientific studies and decision making by resource managers and conservation planners. Since the release of our SDMs, we have seen analyses that incorporate summaries of species applied in the literature^14,15^ and in prioritization efforts^16^. Within each of these efforts, richness values were calculated from SDMs by counting the number of species predicted to be present at each stream reach. In some cases, these efforts used SDMs of all available species to create richness metrics, whereas others used subsets of modeled species specific to their needs, based on traits or conservation status. In all cases, these richness values were paired with spatial datasets that provided additional context and enhanced the relevance to the study or conservation effort.

One recent example was conducted in support of the National Aquatic Connectivity Collaborative^16^ (NACC), where a national collaboration of agencies and organizations helped provide a list of fish richness metrics that would serve as spatial filters to support managers in prioritizing aquatic barriers for removal, restoration, and mitigation. Among the recommended species richness metrics were summaries describing the numbers of migratory species and state-listed Species of Greatest Conservation Need (SGCN) in streams associated with barriers. To ensure usefulness for NACC, these species richness metrics needed to be presented at the spatial resolution of a stream reach, which was not previously available. The project team used our SDMs to calculate richness of migratory species and state-listed SGCN species.

**Fig. 1:**
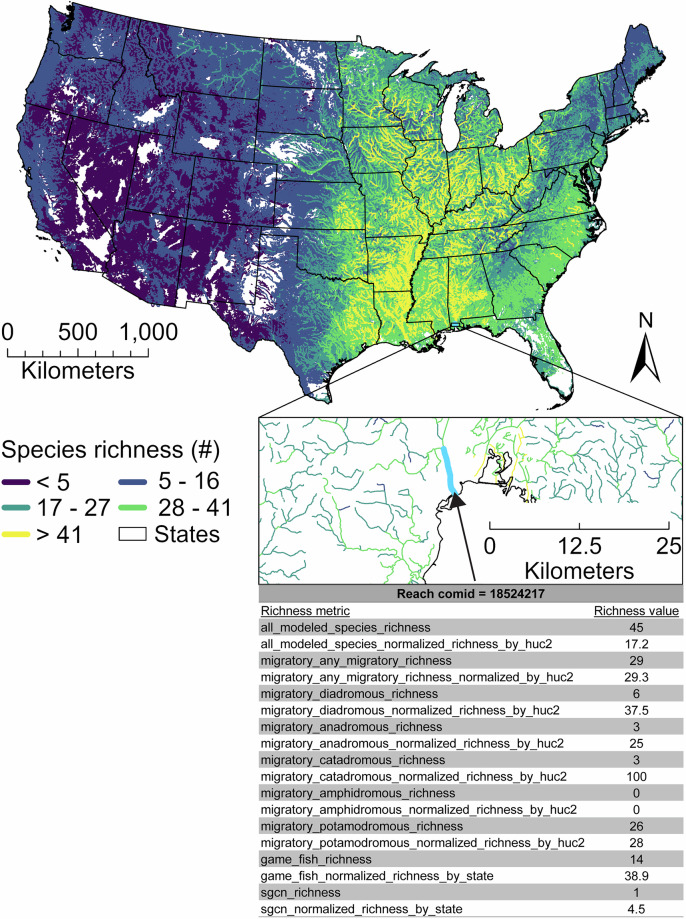
This figure depicts the spatial resolution, extent, and assortment of the fluvial fish richness data. The map of the conterminous U.S. shows an example of how any of the 18 fluvial fish richness metrics can be visualized. In the provided example, the map is symbolized to represent the richness of fluvial fishes based on all available modeled species (n = 415). The map inset depicts the spatial resolution of the dataset, where richness metrics are available at a stream reach scale for each of ~2.3 million stream reaches. Further, the table shows how each stream reach has 18 fluvial fish richness metrics available within the dataset. Spatial data for state boundaries are from the U.S. Census Bureau.

To help inform scientific studies and conservation efforts similar to NACC, we calculated 18 richness metrics using our SDM results (Table 1, Fig. 1). Within these metrics we refer to richness as the number of species that are modeled as present for a given stream reach. Metrics are categorized into four conservation relevant groups described below including (1) *Richness of All Modeled Species*, (2) *Migratory Fish Richness*, (3) *Species of Greatest Conservation Need Richness*, and (4) *Game Fish Richness*.

**Table 1:** Fluvial fish species richness metrics with group representing one of the four conservation relevant groups, the metric source providing the reference of the dataset used to assign metric designations, and the richness metric defining the unit of summary.

Group	Richness Metric	Geographic Normalization*	Metric Source
All Modeled Species	Richness	None	Calculated from Yu *et al*. 2022 v2.0^11^
All Modeled Species	Normalized Richness	HUC2**	Calculated from Yu *et al*. 2022 v2.0^11^
Migratory	Any Migratory Richness	None	Dean *et al*.^25^
Migratory	Any Migratory Normalized Richness	HUC2**	Dean *et al*.^25^
Migratory	Diadromous Richness	None	Dean *et al*.^25^
Migratory	Diadromous Richness	HUC2**	Dean *et al*.^25^
Migratory	Anadromous Richness	None	Dean *et al*.^25^
Migratory	Anadromous Richness	HUC2**	Dean *et al*.^25^
Migratory	Catadromous Richness	None	Dean *et al*.^25^
Migratory	Catadromous Richness	HUC2**	Dean *et al*.^25^
Migratory	Amphidromous Richness	None	Dean *et al*.^25^
Migratory	Amphidromous Richness	HUC2**	Dean *et al*.^25^
Migratory	Potamodromous Richness	None	Dean *et al*.^25^
Migratory	Potamodromous Richness	HUC2**	Dean *et al*.^25^
Game Fish	Richness	None	Stratton *et al*.^26^
Game Fish	Normalized Richness	State	Stratton *et al*.^26^
Species of Greatest Conservation Need	Richness	None	Wellman *et al*.^27^
Species of Greatest Conservation Need	Normalized Richness	State	Wellman *et al*.^27^

Geographic normalization refers to the geographic extent used to convert counts of species into measures of percent of designated species modeled as present.* HUC2 represents a two-digit hydrologic unit code.**

### Richness of all modeled species

*All Modeled Species* is a group of richness metrics derived from all 415 species modeled across the conterminous U.S. (Fig. 1). Metrics in this group show general patterns of richness that can help identify areas across the nation that potentially support high species richness as well as areas with low richness that might represent unique, rare, or less suitable habitats.

### Migratory fish richness

Our second group of metrics called *Migratory Fish Richness*, calculated richness specific to species sharing different migratory behaviors. Patterns in migratory fish richness can help identify areas in river networks where improved connectivity might benefit multiple migratory species, especially when analyzed alongside potential connectivity barriers such as dams and culverts. Understanding which stream reaches are more likely to support a higher richness of species with specific migratory behaviors can help guide and prioritize habitat connectivity efforts such as dam removals, culvert replacements, and fish passage installations.

### Species of greatest conservation need richness

**Fig. 2:**
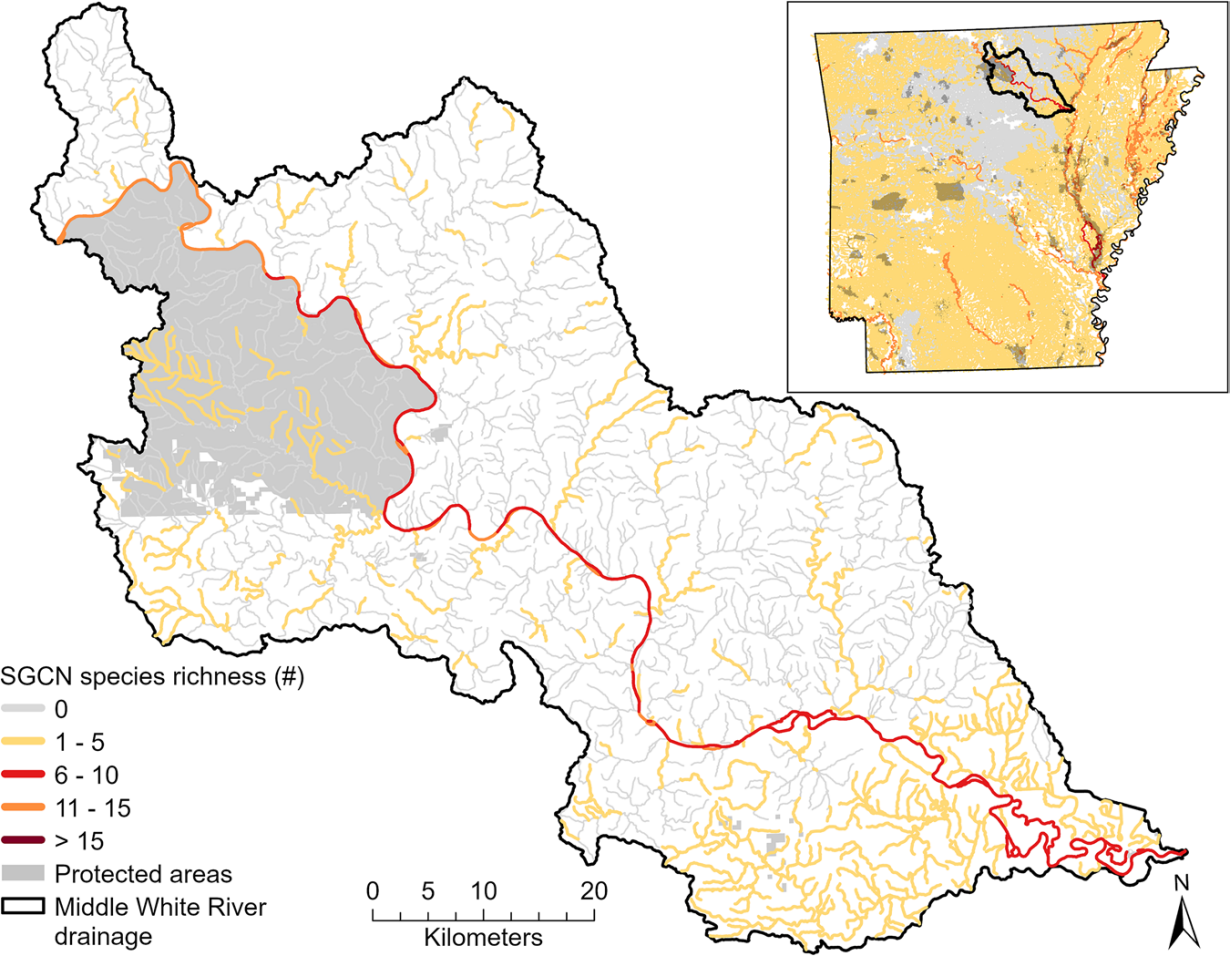
Fluvial fish species richness for modeled Species of Greatest Conservation Need (SGCN) in Arkansas. Protected areas that have a primary goal of biodiversity conservation are overlayed along with the Middle White River watershed boundary to show relative network position of existing protections and SGCN species. A total of 24 fluvial fish species were used to calculate this richness metric. Those species were listed on the Arkansas SGCN list and had an available species distribution model that overlapped the state. Spatial data for state boundaries are from the U.S. Census Bureau, and watersheds from the U.S. Geological Survey^20^.

The third group of richness metrics represent SGCN species. SGCN are designated by each state and the District of Columbia to guide development of State Wildlife Action Plans specific to imperiled species. SGCN fluvial fish richness metrics are calculated for each stream reach based on designations of the 48 states in the conterminous U.S. along with the District of Columbia. States may have different priorities leading to variability in the variety and quantity of designated fluvial fish, and therefore in many cases this group of richness metrics may be most appropriate for within-state applications. Figure 2 illustrates an example that highlights current protected areas and their proximity to predicted richness of SGCN species. In this example, the Protected Areas Database of the United States^17^ documents protected areas that have a primary goal of biodiversity conservation. Protected areas can play a role in controlling impacts to fish habitat in locations directly draining into a stream reach, and in areas upstream of that reach. When investigating impacts of current or proposed protections on vulnerable species, relative network position of existing protections and SGCN species may provide valuable context.

### Game fish richness

The fourth group of richness metrics represent game species, and informs management of species targeted for recreational and consumptive purposes. Game fish richness metrics are calculated for each stream reach based on individual designations of the 48 states in the conterminous U.S. along with the District of Columbia. These metrics can help highlight stream reaches that may support higher numbers of game fish. Similar to SGCN, the game fish designations vary greatly between states so counts of richness may be better suited for analyzing information on a state-by-state basis. To better support use cases that incorporate multiple states, a normalized richness metric was calculated. To normalize game fish metrics, the richness for a stream reach was divided by number of game fish modeled within the stream reach’s corresponding state.

## Methods

### General processing steps

To fully understand the development and utility of the species richness metrics we first describe the primary data sources used to derive the dataset. These richness metrics were derived from SDMs available in version 2.0 of the U.S. Geological Survey (USGS) Aquatic Gap Analysis Project^11,12^. In particular, this database contained 415 fluvial fish distribution models for native species. These models were developed using a boosted regression tree modeling approach to determine likelihood of individual species presence at each of the 2,290,642 stream reaches across the conterminous U.S. These models predict distributions of individual species using fish presence/absence data and 22 natural and anthropogenic landscape variables known to influence stream fish habitat as predictors. Models were spatially constrained by species native ranges as defined by the *Native Ranges of Freshwater Fishes of North America* dataset^18^. For 257 species that lacked native range information, models were constrained by river basins with known species occurrence locations identified in the dataset called *Coarse Range Maps for Fish Species in the Conterminous United States using HUC8s*^19^. Within the coarse range dataset HUC8s refer to 8-digit hydrologic unit codes of the Watershed Boundary Dataset of USGS^20^. These HUC8s map subbasins or medium-sized river basins within the U.S.

Information from the 415 SDMs was used to calculate richness metrics representing the four conservation relevant groups of summaries for each of the stream reaches with SDM results (Table 1). These groups include 1) *Richness of All Modeled Species*, 2) *Migratory Fish Richness*, 3) *Species of Greatest Conservation Need Richness*, and 4) *Game Fish Richness*. To derive richness metrics for these summaries, we first joined tabular information from each source dataset listed in Table 1 to the list of modeled species. Taxonomic serial numbers from the Integrated Taxonomic Information System^21^ are available in each of the source datasets and serve as a unique identifier for discerning each species. The taxonomic serial numbers, retained in our dataset in the field *itis_tsn*, were used to join data across sources, allowing us to then designate if a species contributed to each of the richness metric summaries. Next, for each richness metric in Table 1, a count of species that were designated for that metric and also modeled as present at a given stream reach was calculated. To help control for disproportionate numbers of designated species modeled across geographic footprints, a normalized value was also calculated for richness metrics. The normalization process calculates the percent of all designated species modeled in a geographic extent that are present at a stream reach. For each richness metric within the groups labeled as *All Modeled Species* and *Migratory* (Table 2), values were normalized by two-digit HUCs (*huc2*) that were derived from vector processing unit assignments of stream reaches in the National Hydrography Dataset Version 2.1^13^, which reflect drainage units. For richness metrics within the groups labeled as *Game Fish* and *Species of Greatest Conservation Need*, values were normalized in a similar manner but using state boundaries^22^ (*state_name*). For example, game fish metrics were calculated on state-specific information. The number of designated game fish in states ranged from five in Rhode Island, Utah, and Arizona, to 36 in both Nebraska and Alabama. A richness of 1 in Arizona would be normalized to 20%, whereas a richness of one in Nebraska would be normalized to 2.8%.

Python (version 3.11)^23^ was used to handle all data processing and joins and to calculate all metrics. A repository with specific Python code, a full list of requirements, and additional documentation for each step is available as part of the associated data release^24^.

### Dataset specific steps

We used the North American Freshwater Migratory Fish Database^25^ (NAFMFD) to determine migratory status of individual species. This database is developed from journal articles and scientific reports that describe species as having specific migratory behavior(s). Depending on how behaviors were described in publications, a species could be assigned to discrete migratory behaviors categories such as potamodromous, catadromous, anadromous, diadromous, and amphidromous. Species could also be assigned to multiple migratory behaviors, semi migratory behaviors, suspected migratory behaviors or general migratory behaviors. Due to each species having the option for multiple NAFMFD migratory behavior assignments and the range in discreteness within assignments, we simplified the full suite of NAFMFD migratory behavior assignments into six inclusive metrics including *potamodromous_richness*, *catadromous_richness*, *anadromous_richness*, *diadromous_richness*, *amphidromous_richness*, and *any_migratory_richness*. With this simplification, if any assignment within NAFMFD indicated a species to be characterized in these more inclusive classes it was designated as such for our efforts. For example, NAFMFD assigned the following behaviors to the species *Morone saxatilis* (white bass): *migratory*, *semi-anadromous*, *potamodromous*, and *diadromous_potamodromous*. Our metric development process would include the white bass in the following migratory metrics: *any_migratory_richness*, *anadromous*, *potamodrous*, and *diadromous*.

The sources for both game fish^26^ and SGCN^27^ metrics designate species for each state independently. In other words, some states may designate a species as either SGCN or a game fish or both, whereas another may not. To help handle state-specific information, each stream reach was assigned to an individual state by intersecting stream reach midpoints with state boundaries. When joining information from the source datasets to SDM results, the joins were placed on both species identifier (*itis_tsn*) and state name (*state_name*) to ensure designations were state specific.

## Data Records

The dataset is publicly available in a USGS data release^24^. Fluvial fish richness metrics are available in a geopackage file format, with geospatial information represented in the coordinate reference system of EPSG 5070: NAD83/UTM Zone 15 N. All information is organized in a single geospatial layer representing stream reaches at a 1:100,000 scale, which can be visualized in the map inset in Fig. 1. Fluvial fish richness metrics are available for 2,290,642 stream reaches. We chose to include an additional 400,697 stream reaches that did not contain metric values but allowed for a more complete understanding of connectivity between stream reaches when mapping data. Examples of these cases include flow paths through lakes and reservoirs. When metrics were not available for a stream reach, values were left empty (i.e., null).

Each record in the dataset has a common identifier (*comid*), attributes describing which state (*state_name*) and hydrologic drainage unit (*huc2*) each stream reach overlays, a count of species modeled, and the suite of richness metrics. Field names for richness metrics follow the patterns expressed in Table 2. In total, nine richness metrics, and nine normalized richness metrics are available.

**Table 2:** Patterns used in naming richness metrics.

field type	metric field name
species richness metric naming pattern	{group}_{richness metric}
field name example	game_fish_richness
Normalized modeled metric naming pattern	{group}_{richness metric}_by_{geographic normalization}
Normalized modeled metric naming example	game_fish_normalized_richness_by_state

To communicate which species counted towards each metric, a non-spatial table named *species_metric_designations* is included. Each record in this table represents a species and contains information describing migratory, SGCN, and game fish designations. In addition, common name (*common_name*), scientific name (*scientific_name*), and taxonomic identifier (*itis_tsn*) values from SDMs are provided as a reference. For migratory behaviors, each behavior is represented as a new field, and a matrix of ones and zeros are used to identify if a species was designated or not designated as migratory, respectively. For SGCN and game fish, a separate field is included with a list of states that designated the species for the corresponding metric.

## Technical Validation

These data efforts relied on summarizing previously available datasets. No technical validations were conducted on source datasets beyond those described in their original metadata. Connecting datasets using taxonomic serial numbers helped to minimize potential discrepancies that may be encountered when using species scientific and common names.

To verify metrics that we calculated, we used Python scripts within Jupyter Notebooks^28^ to review new code as it was added, ensuring results met expectations throughout the development phase. Post-development, we examined stream reach values to validate that richness values were within expected ranges. For normalized richness, all values were verified to be within the range of 0 and 100 percent. For all other richness metrics, values were a minimum of 0. For SGCN and game fish metrics, total number of species designated within a state was calculated using the final table *species_designations_for_richness_metrics*. These values were used to ensure the corresponding richness values were equal to or less than these maximum values.

## Usage Notes

These efforts were conducted using readily available data products that provided consistency at the full spatial extent of the conterminous U.S. Although this provides many advantages and opportunities discussed throughout this paper there are a few limitations to consider. When available, the predicted occurrences in this study were limited to species’ native ranges. Because of this, results are not available for species occurrences outside of their native ranges. We used the most recent version of SDMs which includes 415 of ~1,200 native freshwater fish species in the U.S. A current list of species used in these richness summaries can be referenced in the SDM source dataset^11^. Users should be aware that the unmodeled species constitute a large proportion of less common species which have fewer data to produce sufficient modeling results. As new versions of source SDMs become available, we plan to reflect these changes in new versions of fish richness metrics.

## Data Availability

The dataset is publicly available in a USGS data release^24^.

## Code availability

The Python code used to develop richness metrics is provided in the data release^24^.
